# Perioperative Surgical Home Model Improves Outcomes in Crohn's Disease Patients Undergoing Disease-Related Surgery

**DOI:** 10.1155/2020/4293420

**Published:** 2020-07-17

**Authors:** Hang Xu, Danhua Yao, Yuhua Huang, Haining Fan, Yousheng Li

**Affiliations:** ^1^Department of Hepatic Surgery, Fudan University, Shanghai Cancer Center, Shanghai, China; ^2^Department of Surgery, Shanghai Ninth People's Hospital, Shanghai Jiaotong University School of Medicine, Shanghai, China; ^3^Department of Hepatopancreatobiliary Surgery, Affiliated Hospital of Qinghai University, Xining, Qinghai, China

## Abstract

**Background:**

To evaluate Perioperative Surgical Home (PSH) practice model implementation in Crohn's disease (CD) patients undergoing disease-related surgery.

**Methods:**

A retrospective analysis of CD patients requiring disease-related surgery in the Shanghai Ninth People's Hospital was undertaken. Subjects were divided into a non-PSH group consisting of 49 patients (June 2016 to November 2017) and a PSH group consisting of 72 patients (December 2017 until May 2019). Conventional treatment was used for the non-PSH group, while in the PSH group, a standardized pre- and postoperative management routine was employed. The postoperative lengths of stay and incidences of postoperative complications were analyzed.

**Results:**

There were no significant differences in demographics, reasons for surgery, preoperative BMIs, and preoperative hemoglobin between the two groups (*P* > 0.05). The overall incidence of complications in the PSH group was dramatically lower than that in the non-PSH group (26.4% vs. 44.9%, *P* = 0.035). In the PSH group, postoperative length of stay was significantly shorter than that in the non-PSH group (11.5 ± 5.7 vs. 9.0 ± 6.8, *P* < 0.001).

**Conclusions:**

The PSH conditioning routine in CD patients undergoing disease-related surgeries suggests a trend of fewer postoperative complications and shorter lengths of hospital stay. The PSH model may have clinical advantages when applied to CD patients.

## 1. Introduction

Crohn's disease (CD) is an inflammatory bowel disease of unknown etiology that can occur in any part of the gastrointestinal tract. The incidence of CD in China is steadily increasing [1]. CD patients often require surgical treatment for bowel strictures, intestinal fistulas [2], gastrointestinal hemorrhage, or other complications that cannot be controlled with medication. Secondary to the nature of CD and the approaches employed in its management, the incidence of malnutrition in hospitalized CD patients is approximately 75% [3], while the incidence of malnutrition in patients undergoing surgery for CD is even higher. In addition to nutritional risks, CD patients experience intestinal inflammation and mesenteric fat edema [4]. Therefore, the postoperative infection and mortality rates in CD patients are high, and these patients require greater rehabilitation and support prior to surgery.

Surgery is the mainstay treatment for CD complications [5]. It is estimated that half of CD patients will require surgery within 10 years of diagnosis and approximately 80% will require surgery at some point in their lives [6]. Because the risk of both postoperative complications and postoperative recurrence is high, nearly half of all CD patients will require a second surgery or even multiple surgeries. Repeated surgery, however, increases the risk of short bowel syndrome [7].

To improve the clinical course of our patients, we have introduced the principles of Perioperative Surgical Home (PSH) management into our surgical practice and formulated a set of clinical guidelines suitable for CD patients undergoing disease-related surgery. Compared with cancer patients, the surgical timing for CD patients is more flexible. This is why PSH is suitable for surgical treatment of CD. The patients included in this study were all undergoing elective surgery, and no patient required emergency surgery.

In surgical management of CD, the early involvement of a multidisciplinary team should be considered [8]. So, our unit is led by general surgeons in combination with faculty from the departments of anesthesiology, gastroenterology, imaging, and nutrition. Since December 2017, we have officially incorporated the PSH management model into our clinical practice.

## 2. Methods

This study was approved by the Institutional Review Board of the Shanghai Ninth People's Hospital.

**Figure 1:**
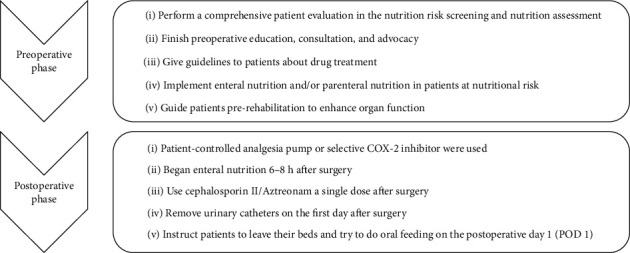
The key additional preoperative and postoperative elements of the Perioperative Surgical Home model at the Shanghai Ninth People's Hospital.

The 2 study groups were derived from 2 sequential 18-month time periods. To reduce selection bias, we collected outcome data of all consecutive cases, which underwent CD disease-related surgery from June 2016 to May 2019 in the Shanghai Ninth People's Hospital. Patients with acute intestinal obstruction, free peritoneal perforation, or complications requiring emergency surgery were excluded from the study. Since the PSH management model was introduced into our clinical practice in December 2017, all patients treated between June 2016 and November 2017 are considered the non-PSH group. And all patients treated from December 2017 until May 2019 are considered to be the PSH group. The treatment of patients in the PSH group was carried out using clinical guidelines based on the PSH model of pre- and postoperative management. Treatment protocols employed in patients in the non-PSH group were based upon the judgment of the surgeon. The PSH protocol is described in Figure 1.

## 3. PSH Details

Once the decision was made to operate on a patient, nutrition risk screening and nutrition assessment were performed. Laboratory tests including albumin and hemoglobin were performed and body mass index (BMI) calculated to assess patient nutritional status. Routine movement assessment was carried out, including a hand grip strength test and a 6-minute walk test (6-MWT). The focus of the PSH model is optimization of preoperative drugs (in terms of type and schedule), nutritional status, and physical condition. PSH-derived prerehabilitation has been noted to increase patient tolerance of surgery, promote postoperative recovery, and shorten the length of hospitalization [9].

### 3.1. Preoperative Education, Consultation, and Advocacy

At the time of outpatient consultation and after the decision to operate was made, patients were placed on the PSH protocol. The outpatient physician assessed whether the patient could tolerate surgery by evaluating nutritional status and hemoglobin levels and reviewed current drugs taken assessing whether the patient needed to discontinue or change medications. The patients and their relatives were informed of the detailed process of inpatient treatment, including the estimated length of surgery, preparations required prior to surgery, selection of surgical protocol, potential postoperative complications, corresponding measures to reduce surgical risk, and the expected length of stay. Patients were informed of matters relevant to PSH protocol implementation. After the patient agreed to undergo PSH protocol management, consent specific to the protocol was obtained.

### 3.2. Drug Treatment

In the PSH group, patients were asked to let the physician know what drugs they were taking and then given guidance as to what adjustments were needed to be made. Specifically, prednisone and/or prednisolone were discontinued for 2 weeks prior to surgery. If hormones could not be rapidly discontinued, the patients were required to reduce the dose of hormones to 20 mg/d or <0.5 mg/kg/d. No biological preparations were used during the 2 weeks prior to surgery. Because CD patients have a high incidence of anemia [10], patients with a history of anemia (defined as a hemoglobin level of 80–100 g/L) were required to take an oral iron supplement. Patients with hemoglobin counts lower than 80 g/L received a recommendation to go to a community hospital for intravenous iron supplementation. The methods of iron supplementation employed were based on the guidelines from “European Consensus on the Diagnosis and Management of Iron Deficiency and Anaemia in Inflammatory Bowel Diseases” [11]. Mandatory requirements for drug treatment were not made for the non-PSH group. For example, we did not require patients to discontinue steroids or biologics in the 2 weeks before surgery.

### 3.3. Nutritional Support

During outpatient consultation, a specialist employed the nutrition risk screening tool NRS2002 [12]. Patients at nutritional risk underwent additional evaluation of their nutritional status employing the Patient-Generated Subjective Global Assessment (PG-SGA) [13] followed by preoperative nutritional support for 10–14 days. The preoperative nutritional support strategy was tailored to the actual condition of the patient, including compliance and occupation. Entire enteral nutrition (EEN) is the best choice. Partial enteral nutrition (PEN) is another way. Besides, we also choose oral nutritional supplements (ONS). If a patient had complete intestinal obstruction, an ileus tube was placed via endoscopy, followed by fasting, inhibition of digestive juice secretion, and total parenteral nutrition support with the goal being to decrease the degree of intestinal tract expansion facilitating stage 1 anastomosis during surgery. For the non-PSH group, patients did not receive nutritional support.

### 3.4. Prerehabilitation of Organ Function

Patients underwent grip strength screening during outpatient consultation. Patients with a grip strength below the normal range also underwent the 6-MWT with baseline values recorded. Following that, health education was provided to patients to help them carry out endurance training, including aerobic and anaerobic exercises. Aerobic exercise included either 30 min of running or bicycle riding 4 times a week. Anaerobic exercise involved 30 min of strength training. Patients in the non-PSH group did not do these.

### 3.5. Surgery

Surgeries were performed by the same surgical team. We gave cephalosporin II or Aztreonam 30 min [14, 15] before surgery. In patients with stenosis and internal fistula, if the anastomotic segment in the small intestine exceeded 6 cm in diameter, staged surgery was carried out. First, the segment of the intestine with lesions was resected and an enterostomy was performed. At a later date, the stoma was reduced. If the small intestine requiring anastomosis was either not expanded or only mildly expanded, the intestinal segments with lesions were directly resected and anastomosis was carried out in a single operation. If an ileus tube had been placed, the tube was removed at this time.

### 3.6. Postoperative Medication

Before surgery, the anesthesiologist and the patient discussed the method to be used for postoperative analgesia. Two possible approaches were presented. The first involved a patient-controlled analgesia pump. Alternatively, a selective COX-2 inhibitor could be administered intravenously by the staff. No opioids were employed postoperatively unless a patient's pain score was greater than 7. We used parecoxib (40 mg Q 12 h) until patients started eating. They were then transitioned to celecoxib 20 mg Q 12 h by oral administration. 6–8 h postoperation, we began enteral nutrition in order to enhance motility/contractility of the gastrointestinal tract [16]. For antibiotics, we used cephalosporin II or Aztreonam, which was administered in a single dose after surgery. In this part, we did the same things approximately between the two groups.

### 3.7. Postoperative Rehabilitation Guidance

Patients who did not undergo bladder repair and had no preoperative bladder fistula all had their urinary catheters removed on the first day after surgery. Also, on the first postoperative day, patients were instructed to leave their beds and began oral feeding or oral enteral nutrition to stimulate recovery of intestinal function. Once confirmation had been made that there was no anastomotic fistula, the abdominal drainage tubes were removed. For the non-PSH group, patients did not receive mandatory requirements to leave their beds.

## 4. Statistical Analysis

Demographic data collected included previous medical history, surgical history, preoperative hemoglobin levels, preoperative albumin levels, length of hospital stay, postoperative complications, and perioperative deaths. Statistical analysis was applied to the length of stay, postoperative complications, and deaths. Postoperative complications were analyzed using the Clavien-Dindo grading system for surgical complications (grades I–V) [17], including the overall incidence of complications and the incidence of minor or major complications.

SPSS 20.0 data analysis software was used to process experimental data and carry out the statistical analysis. Continuous variables were compared using the independent sample *t*-test or Mann-Whitney *U* test. Categorical data was compared using the chi-squared test or Fisher's exact test. A difference with *P* < 0.05 was considered to be statistically significant.

## 5. Results

Retrospective analysis of the records of a single institution from June 2016 to May 2019 identified a total of 121 patients that satisfied the study inclusion criteria. Forty-nine patients (June 2016 to November 2017) comprised the non-PSH group and 72 patients (December 2017 until May 2019) formed the PSH group.  Table 1 shows the demographic information for the 2 patient groups. There were no statistically significant differences in sex, age, BMI, hemoglobin, or number of previous abdominal surgeries between the 2 groups of patients (*P* > 0.05). Not unexpectedly, the preoperative albumin levels of the patients in the PSH group were significantly higher than those in the non-PSH cohort (34.4 ± 3.6 vs. 35.8 ± 2.7, *P* < 0.05).

Table 2 shows the surgeries performed. There were no significant differences in the diverting stoma rates (24.5% vs. 19.4%, *P* = 0.507).  Table 3 shows the postoperative complications among the groups. There were no grade V complications in either patient group.  Table 4 shows the postoperative recovery status for the 2 groups. There were no significant differences in the incidence of minor complications (grades I and II) or major complications (grades III–V) between the 2 groups. However, the overall incidence of complications was lower in the PSH group than in the non-PSH group (44.9% vs. 26.4%, *P* = 0.035). The postoperative lengths of stay (11.5 ± 5.7 vs. 9.0 ± 6.8, *P* < 0.001) were also significantly shorter in the PSH group.  Table 5 shows the pre- and postoperative medications we used in the PSH group.

## 6. Discussion

Perioperative Surgical Home management is a patient-centric, multidisciplinary, collaborative, and external-internal integrative medical model that begins with surgical preparation and continues until complete recovery. Its core tenet is that PSH management continues throughout the entire treatment process. In addition, the process is patient-centric, as the patient and physician jointly determine the regimen. The PSH model was implemented with the hope that perioperative treatment quality and satisfaction could be improved, and medical resource allocation would be optimized, leading to reduced medical costs, shortened hospital stays, and lowered readmission rates. The concept of PSH management was officially proposed in 2014 by the American Society of Anesthesiologists [18]. There are four central aspects to PSH management—(1) the team providing medical services is led by the anesthesiologist, while surgeons, internists, physiotherapists, nurses, laboratory technicians, radiologists, pharmacists, information technicians, and social workers jointly participate; (2) the PSH protocol spans the entire treatment period from the time a patient decides to undergo surgery (prior to actual hospitalization) until the time the patient has completely recovered and has been discharged; (3) the PSH protocol engages with the family, rehabilitation hospital, nursing home, and community clinic; and (4) the PSH protocol is patient-centric, allowing the patient to participate in decision-making throughout the process. Together, these tenets not only improve the quality of the medical treatment but also provide humanistic care [19], because communication and psychological assistance with CD patients are important [20].

Since the concept of PSH was proposed, many departments have implemented this model in clinical work, with acceptance by both doctors and patients. The implementation of the PSH management protocol can improve the prognosis of patients who undergo total hip or knee arthroplasty, in part, by significantly decreasing the rate of surgical delays, decreasing the incidence of postoperative complications, and reducing overall medical costs [21]. In pediatric patients undergoing adenoidectomy, use of the PSH management approach significantly shortened the length of stay and reduced hospitalization costs, while not increasing the 30-day readmission rate [22].

CD is a chronic, recurrent inflammatory bowel disease. Due to the long-term nature of the disease, CD patients typically have a good understanding of their disease. Our hospital is a renowned surgical center in China. In contrast with other CD centers, most of our patients are suffering from more severe CD and require surgical intervention. As a result, our CD patients usually demonstrate good compliance with prescribed therapies. Therefore, Li introduced the PSH management protocol, which is suitable for CD patients undergoing surgery, into our unit, the first to implement this protocol in China. Due to the good compliance, we finished this program successfully without too much difficulty. Secondary to practice patterns in China, the staff that are the first to contact patients are usually internists. However, in our institution, PSH implementation was led by general surgeons, in conjunction with a multidisciplinary treatment team of anesthesiologists, internal medicine specialist, radiologists, nutritionists, and laboratory personnel. In fact, we were not the first to consider surgeon-led PSH teams. This approach has been reported by others [23] in the management of ophthalmology, head and neck cancer, gynecology and obstetrics, and general surgery patients.

Oral and intravenous iron supplementation is important for CD patients with confounding anemia [24]. It is important to point out that the patients in the PSH group underwent preoperative hemoglobin assessment and anemic patients received iron supplementation or erythropoiesis stimulation before surgery. But there are no statistical differences between the two groups in the preoperative hemoglobin levels. The reason can be attributed to the hemoglobin level which usually rises after 2 weeks of oral iron.

Perioperative nutritional support is a well-known adjunct to many surgical diseases, including Crohn's [25, 26]. Enteral nutrition can improve nutritional status, reduce inflammation, promote mucosal healing, reduce intestinal permeability, and change intestinal bacterial flora. These effects can improve the clinical symptoms of CD patients, promoting CD remission [27]. In the present case, patients in the PSH group that underwent 10–14 days of nutritional support prior to surgery (based on the guidelines from the American Society for Parenteral and Enteral Nutrition (ASPEN) [28]) showed a significant difference in preoperative albumin compared to non-PSH patients. Nutritional support likely accounted for elevated preoperative albumin levels in patients from the PSH group as compared to those of the non-PSH group. However, preoperative BMIs have no statistical differences. The reasons for this are not known but may be related to the overall length of nutritional support. In our study, diverting stoma rates are not reduced. This may be because performing an ostomy is determined by the severity of the patient's disease.

A recent study shows that wound infection, intra-abdominal abscess, and anastomotic leak are the most common complications after intestinal resection with ileocolonic anastomosis in Crohn's disease, about 24.2% of all patients [29]. In our study, the complication rate of 26.4% seems high, even after PSH. This may be due to our use of the Clavien-Dindo grading system for surgical complications. And the majority of complications are minor complications (fever, wound infection, diarrhea, blood transfusion, total parenteral nutrition, and early postoperative bowel obstruction). Another research shows that 33.1% of their patients had postoperative complications [30]. In a recent study, a similar enhanced recovery after surgery (ERAS) protocol is reported to be associated with decreased rates of postoperative surgical site infection (SSI), ileus, and anastomotic leak [31]. In our research, minor complications, major complications, and each complication did not decrease in the PSH group. But the overall complication rate is significantly reduced in the PSH group. This bias may be due to the small sample size.

ERAS refers to the application of a series of optimized perioperative treatment measures with evidence-based medical evidence to reduce the perioperative psychological and physiological stress responses of surgical patients, so as to achieve the purpose of rapid recovery. Studies have confirmed that the use of ERAS in CD patients requiring surgery is safe and effective [32, 33]. In our PSH model, there are some aspects similar to ERAS. They all achieve rapid rehabilitation through a series of optimization measures. When we designed the PSH protocol, we integrated a lot of evidence-based medical evidence and clinical experience. For example, the use of steroids in patients with CD increases the risk of infectious complications [34], and nutritional support is important to the perioperative care of patients with CD [35]. To relieve the disease, steroids or/and biologics are used for some patient wounds. In the PSH protocol, we require this part of patients to reduce the dose of hormones and not use biologics 2 weeks before surgery. And we put nutritional support as a very vital point in the PSH model. Different from ERAS, the PSH model emphasizes taking patients, diseases, and doctors in multiple disciplines as a whole. It runs through the entire process from decision surgery to recovery. Compared to ERAS, the PSH model extends space into the home and time before admission. In this way, patients can participate in the management of the disease, deepen their understanding of the disease, eliminate the fear of surgery, and achieve the purpose of rapid recovery.

The present study has a number of limitations. First, it was a retrospective analysis with relatively small cohort sizes. Also, being retrospective in nature, patient selection was possibly unintentionally biased. Another study limitation was that it encompassed the experience of only a single institution. Further, treatment assignment was not randomized. Nonetheless, there were no statistically significant differences found in demographics or reasons for surgery between the 2 groups of patients, while the PSH group was noted to fare better in several surgery-related categories compared to the non-PSH group. Randomized controlled clinical trials with large sample sizes are needed to further validate the implementation results of the PSH model in CD patients undergoing disease-related surgery.

## 7. Conclusion

In summary, this is the first report on the PSH model in CD patients undergoing disease-related surgery. As a preliminary report, this study demonstrated that this routine has a trend of fewer postoperative complications and shorter lengths of hospital stay. Finally, PSH may be one way to solve the problem of providing quality treatment in constrained medical resource environments in China.

## Figures and Tables

**Table 1:** Demographic and clinical characteristics of study cohorts.

	Non-PSH	PSH	*P* value
Number of patients (%)	49 (40.5)	72 (59.5)	
Male, *n* (%)	32 (65.3)	52 (72.2)	0.418
Age (y) (±SD)	35.9 ± 10.4	37.3 ± 10.2	0.239
BMI (kg/m^2^) (±SD)	17.3 ± 1.6	17.7 ± 1.4	0.111
Preoperative albumin (g/L) (±SD)	34.4 ± 3.6	35.8 ± 2.7	0.016^∗^
Preoperative hemoglobin (g/L) (±SD)	105.7 ± 18.7	110.7 ± 14.6	0.107
Previous abdominal surgery, *n* (%)	6 (12.2)	12 (16.7)	0.502
Smoking status, *n* (%)	0	1 (0.01)	0.409
Drinking status, *n* (%)	0	0	

Non-PSH: non-PSH group; PSH: PSH group. ^∗^*P* < 0.05 for Student's *t*-test comparison between the non-PSH group and the PSH group.

**Table 2:** Surgical therapies and rationale in treatment cohorts.

	Non-PSH group (*n* = 49)	PSH group (*n* = 72)	*P* value
Surgical strategy			0.248
Laparotomy, *n* (%)	36 (73.5)	45 (62.5)	
Laparoscope, *n* (%)	13 (26.5)	27 (37.5)	
Surgical procedures			0.438
Enteroenterostomy, *n* (%)	5 (10.2)	13 (18.1)	
Enterocolostomy, *n* (%)	30 (61.2)	40 (55.6)	
Colocolostomy, *n* (%)	2 (4.1)	5 (6.9)	
Diverting stoma, *n* (%)	12 (24.5)	14 (19.4)	0.507
Reason for surgery			0.885
Internal fistula, *n* (%)	15 (30.6)	17 (23.6)	
External fistula, *n* (%)	4 (8.2)	6 (8.3)	
Obstruction, *n* (%)	24 (49.0)	37 (51.4)	
Obstruction+internal fistula, *n* (%)	3 (6.1)	7 (9.7)	
Internal fistula+external fistula, *n* (%)	3 (6.1)	4 (5.6)	
Obstruction+external fistula, *n* (%)	0	1 (1.4)	

**Table 3:** Postoperative complication status in patient cohorts (Clavien-Dindo grading system).

	Non-PSH group (*n* = 49)	PSH group (*n* = 72)	*P* value
Grade I			
Fever, *n* (%)	2 (4.1)	2 (2.8)	0.695
Diarrhea, *n* (%)	2 (4.1)	3 (4.2)	0.982
Wound infection, *n* (%)	3 (6.1)	3 (4.2)	0.628
Grade II			
Blood transfusions, *n* (%)	3 (6.1)	2 (2.8)	0.366
Total parenteral nutrition, *n* (%)	2 (4.1)	1 (1.4)	0.352
Ileus, *n* (%)	1 (2.0)	2 (2.8)	0.799
Grade III			
Gastrointestinal bleeding, *n* (%)	3 (6.1)	2 (2.8)	0.366
Intra-abdominal bleeding, *n* (%)	1 (2.0)	0	0.225
Pleural effusion, *n* (%)	1 (2.0)	1 (1.4)	0.783
Ascites, *n* (%)	0	1 (1.4)	0.409
Anastomotic leakage, *n* (%)	3 (6.1)	2 (2.8)	0.366
Grade IV			
Sepsis, *n* (%)	1 (2.0)	0	0.225
Grade V			
Death	0	0	

If a patient experienced 2 or more complications, the complication with the highest grade was used for statistical analysis.

**Table 4:** Postoperative recovery status between non-PSH and PSH patients.

	Non-PSH group	PSH group	*P* value
Postoperative length of stay (days)	11.5 ± 5.7	9.0 ± 6.8	<0.001^a^
Incidence of postoperative complications, *n* (%)	22 (44.9)	19 (26.4)	0.035^b^
Grades I and II, *n* (%)	13 (26.5)	13 (18.1)	0.265
Grades III–V, *n* (%)	9 (18.4)	6 (8.3)	0.100

^a^*P* < 0.05 for Mann-Whitney *U* test between the non-PSH group and the post-PSH of the PSH group. ^b^*P* < 0.05 for chi-squared test between the non-PSH group and the PSH patients.

**Table 5:** Pre- and postoperative medications.

	Preoperation	Postoperation
Nutritional therapy		
Enteral nutrition	EEN/PEN/ONS	Started at 6–8 h postoperation
Parenteral nutrition	If necessary	If necessary
Analgesia		
Parecoxib	Not used	40 mg Q 12 h
Celecoxib	Not used	20 mg Q 12 h
Antibiotic		
Prophylactic	Cephalosporin II/Aztreonam	Cephalosporin II/Aztreonam
Therapeutic		Depended on susceptibility testing

In the PSH group, we used these medications as a routine. We also used some others depending on each patient. EEN: entire enteral nutrition; PEN: partial enteral nutrition; ONS: oral nutritional supplements.

## Data Availability

The datasets used or analyzed during the current study are available from the corresponding author on reasonable request.
